# Methicillin-Resistant *Staphylococcus aureus* Prostatic Abscess in a Liver Transplant Recipient

**DOI:** 10.1155/2014/854824

**Published:** 2014-10-20

**Authors:** Tanima Jana, Jorge D. Machicado, Giovanni E. Davogustto, Jen-Jung Pan

**Affiliations:** ^1^Department of Internal Medicine, University of Texas Medical School at Houston, Houston, TX 77030, USA; ^2^Division of Gastroenterology, Hepatology and Nutrition, Department of Internal Medicine, University of Texas Medical School at Houston, Houston, TX 77030, USA

## Abstract

Prostatic abscesses are usually related to gram-negative bacilli. However, methicillin-resistant *Staphylococcus aureus* (MRSA) has emerged as a substantial cause of prostatic abscesses in recent years. Herein, we report the case of a 31-year-old man with a history of orthotopic liver transplantation 10 years ago who presented with acute onset dysuria and abdominal pain and was diagnosed with a MRSA prostatic abscess. To our knowledge, this is the first case describing a prostatic abscess in a liver transplant recipient and the first reporting MRSA as the causative organism of a prostatic abscess in a solid organ transplant recipient.

## 1. Introduction

A prostatic abscess is a rare entity, usually caused by organisms from the family Enterobacteriaceae or genus* Enterococcus*. Although* Staphylococcus aureus* infection is typically associated with skin diseases, it has emerged as a substantial cause of prostatic abscesses in recent years [1].* S. aureus*, a major cause of infection among solid organ transplant recipients, occurs most commonly within the first 2 months following liver transplantation and is associated with a high morbidity and mortality [2]. Herein, we report the case of a 31-year-old man with a history of orthotopic liver transplantation 10 years ago, chronic immunosuppression, and ulcerative colitis, who presented with acute onset dysuria and abdominal pain. He was ultimately found to have a prostatic abscess and bacteremia caused by methicillin-resistant* Staphylococcus aureus* (MRSA). To the best of our knowledge, this is the first case describing a prostatic abscess in a liver transplant recipient and the first reporting MRSA as the causative organism of a prostatic abscess in a solid organ transplant recipient.

## 2. Case Presentation

**Figure 1:**
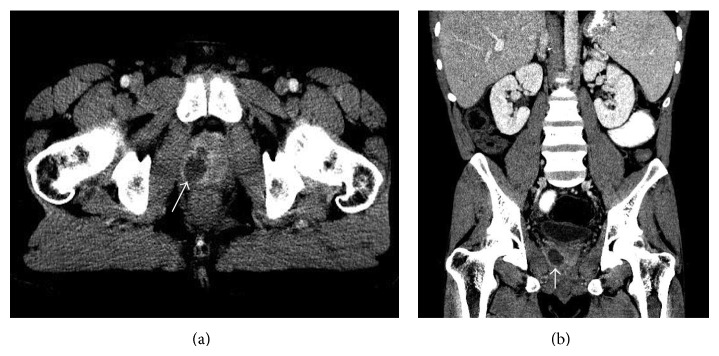
Axial (a) and coronal (b) views of CT abdomen and pelvis revealing a 3.0 cm × 1.9 cm prostatic abscess (arrow).

**Figure 2:**
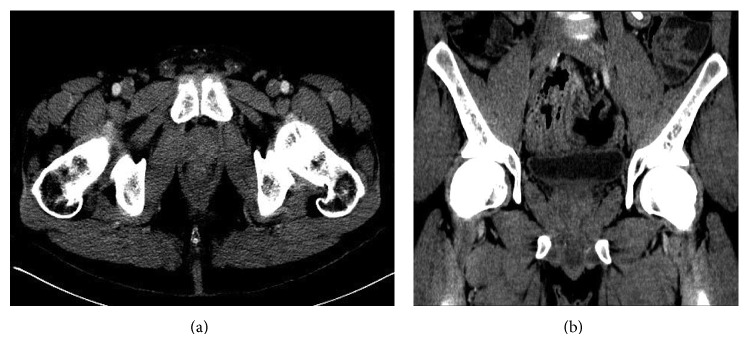
Axial (a) and coronal (b) views of CT abdomen and pelvis with contrast after completion of 6 weeks of intravenous antibiotic therapy showing resolution of prostatic abscess.

A 31-year-old African-American man underwent orthotopic liver transplantation 10 years ago for primary sclerosing cholangitis (PSC). He had moderate acute cellular rejection five months after the transplant but had no history of any transplant-related infections. He presented to the emergency department with a one-week history of lower abdominal pain and dysuria. His past medical history was pertinent for ulcerative colitis and type 2 diabetes mellitus. He had been hospitalized multiple times in the past year for abdominal pain. Extensive investigation had shown partial small bowel obstruction caused by intussusception that was treated conservatively, in addition to chronic elevation of total bilirubin, alkaline phosphatase, and transaminases attributed to possible recurrence of PSC in the hepatic graft. At the time of suspicion of possible PSC recurrence, his prednisone dosage had been increased and later tapered when liver function studies improved. He had last been admitted to the hospital 2 weeks earlier for diarrhea and fever, diagnosed with* Clostridium difficile* infection (CDI), and discharged 10 days prior with oral vancomycin. At the time of current presentation, his immunosuppression regimen included tacrolimus 1 mg twice daily, mycophenolate mofetil 500 mg twice daily, and prednisone 5 mg daily. He reported temporal insertion of indwelling urinary catheters during some of his previous hospital admissions for management of small bowel obstruction. Otherwise, he denied any intravenous (IV) drug use, history of urethral instrumentation, or chronic indwelling urinary catheters. His vital signs on admission revealed blood pressure 134/68 mmHg, heart rate 80 beats per minute, 20 respirations per minute, and temperature 98.5°F. On physical exam, his abdomen was soft with mild tenderness to palpation in the left lower quadrant. Rectal exam revealed a normal-sized, painless prostate with fluctuation at the right base. Laboratory investigation showed white blood cell count of 22,600/*μ*L (94% neutrophils, 2% lymphocytes, 1% bands), hemoglobin 12.3 g/dL, and platelet count 309,000/*μ*L. Biochemistry tests revealed creatinine 1.2 mg/dL, alanine aminotransferase 199 U/L, aspartate aminotransferase 157 U/L, alkaline phosphatase 719 U/L, and total bilirubin 18.3 mg/dL (direct bilirubin 15.6 mg/dL), which were unchanged compared to previous laboratory results. Human immunodeficiency virus (HIV) antibody was negative. Urinalysis showed 11–20 white blood cells/high power field (HPF), 0–2 red blood cells/HPF, few bacteria, and no casts. Contrast-enhanced computed tomography (CT) of the abdomen and pelvis revealed multiple stable areas of intussusception and a 3.0 cm × 1.9 cm prostatic abscess (Figures 1(a) and 1(b)). We empirically treated for infection with IV ceftriaxone and vancomycin. MRSA was isolated from both urine (<10,000 CFU/mL) and 2 sets of blood cultures (aerobic and anaerobic bottles) obtained upon admission. Cultures were sensitive to daptomycin, clindamycin, gentamycin, rifampin, tetracycline, levofloxacin, trimethoprim-sulfamethoxazole, and vancomycin (MIC < 0.5). Both transthoracic and transesophageal echocardiography were negative for vegetations. Based on his recent hospitalizations, he was diagnosed with health-care associated MRSA bacteremia. Detailed review of his hospital records revealed that colonization with MRSA had been detected 2 weeks prior in a nasal swab sample and treated accordingly with topical chlorhexidine and mupirocin. A final diagnosis of MRSA prostatic abscess with bacteremia was made. Due to his immunosuppression and bacteremia, it was decided that he was not a candidate for oral antibiotics. IV vancomycin was continued while ceftriaxone was stopped. We opted for conservative management in this case, which showed rapid improvement of symptoms and inflammatory response. Therefore, abscess aspiration was not deemed necessary. We also continued oral vancomycin to complete 14 days of treatment for CDI. The same dosage of immunosuppressant medications was continued, with the exception of tacrolimus, which was held due to acute kidney injury. Leukocytosis resolved at day 3 while symptoms started to improve at day 5. Repeat blood cultures at day 4 were negative. Urine cultures failed to grow any organism at day 11 of active therapy. His creatinine rose to 1.6 mg/dL and IV vancomycin was switched to daptomycin on day 12 of treatment. He was discharged from the hospital at day 22, with instructions to continue daptomycin for a total of 6 weeks of antibiotics. During a follow-up clinic appointment at 8 weeks after completion of antibiotics, his symptoms were resolved, cultures were negative, and repeat CT of the abdomen and pelvis showed complete resolution of the prostatic abscess (Figures 2(a) and 2(b)).

## 3. Discussion

Prostatic abscess is most commonly caused by gram-negative bacilli, primarily* Escherichia coli*, (60–80% of cases), although* Neisseria gonorrhoeae* was the most common pathogen in the past (75% of cases in the preantibiotic era) [3, 4].* Staphylococcus aureus* is an uncommon cause of prostatic abscess but it has been reported more commonly in recent years. We searched the literature from inception through August 2014 on PubMed and Ovid MEDLINE to identify the cases of prostatic abscess caused by* S. aureus*. The databases were searched using the keywords “*Staphylococcus aureus*” OR “methicillin-resistant* Staphylococcus aureus*” OR “MRSA” AND “prostatic abscess” OR “prostate abscess” OR “prostatic infection” OR “prostate infection.” We found 33 documented cases of* S. aureus* prostatic abscess (Table 1), 21 of which were found to be MRSA, 3 methicillin-sensitive* Staphylococcus aureus *(MSSA), and 9 in which susceptibilities were not available. Of the 21 MRSA cases, 20 (95%) involved patients with predisposing conditions, 18 (86%) fell within the 40–60-year-old age group, and 16 (76%) had associated bacteremia. Predisposing history included immunocompromised conditions (e.g., diabetes mellitus, HIV, immunosuppressive drugs), IV drug use, recent genitourinary infection, chronic or recent indwelling urinary catheter, and preexisting prostatic disease, such as benign prostatic hypertrophy. Additional risk factors for prostatic abscess described in the literature include chronic dialysis, prostate biopsy, chronic bacterial prostatitis, and obstructive uropathy [4–7]. In the case of our patient, he was colonized by MRSA most likely due to his multiple hospital admissions and was receiving immunosuppressant agents for liver allograft protection. In addition, he had been admitted multiple times for partial small bowel obstruction during the last year and undergone temporal indwelling urinary catheter insertions.

Common symptoms of a prostatic abscess include fever, dysuria, increased urinary frequency, acute urinary retention, perineal pain, back pain, and hematuria [3, 7]. Many of these symptoms might be absent in immunosuppressed subjects, and thus a prostatic abscess should be suspected in these patients if found with MRSA bacteriuria or MRSA bacteremia of unknown source. MRSA bacteriuria is rare, with a study from a single institution in Rochester, Minnesota, describing it in 0.5% of 17,085 urine cultures obtained over two years [8]. Risk factors associated with MRSA bacteriuria in this study included recent urinary catheter use, recent healthcare exposure, history of genitourinary surgery, presence of comorbidities, and increasing patient age. Identification of the source of bacteriuria was not described in the study, but it was effectively treated as a urinary tract infection [8]. Clinicians should be reminded that the differential diagnosis after detecting MRSA in the urine should include pyelonephritis, renal abscess, prostate abscess, endocarditis, skin infection, or staphylococcal bacteremia originating from another site [3, 7, 9]. MRSA bacteremia can be the origin or the consequence of a prostatic abscess. In our case, further investigation to identify the origin of the bacteremia, including careful history, physical examination, and echocardiogram, was negative except for the presence of the prostatic abscess.

Imaging studies obtained by CT and transrectal ultrasound (TRUS) are useful in the diagnosis of prostatic abscesses. There are no studies that have described superiority of any of these techniques over the others. TRUS is often used initially and can show one or more hypoechoic areas with well-defined and thick walls containing fluid. However, it may be inconclusive in the initial stages of abscess formation [3, 10]. Additionally, it may cause pain to patients and abscess size may be altered based on the angle of TRUS. CT is not painful, has less operator dependence, and can detect spread of infection to nearby organs [3]. In addition, CT offers better characterization of the lesion and can differentiate an abscess from a cyst, granuloma, or malignancy [4]. There is no consensus regarding which imaging modality to use, and some authors advocate starting with TRUS, as one study showed 100% accuracy in diagnosis of prostate abscesses [11].

While it is widely agreed that prompt treatment is crucial to prevent morbidity and mortality, there are no randomized clinical trials or expert consensus statements that recommend conservative management, percutaneous aspiration, or surgical incision and drainage as the particular treatment strategy of choice [3, 4, 7]. Conservative management is limited to antibiotics targeted against the organism found in the cultures. If an additional intervention is decided, it depends on local experience to decide between transurethral resection of prostate (TURP) and transperineal percutaneous drainage. A 2012 retrospective study from Korea [3] described 52 patients with prostatic abscess of any etiology, where 11 received conservative treatment, 23 underwent TURP, and 18 had transperineal aspiration. The average length of hospital stay was 17.5 days. Of the 18 who underwent needle aspiration, hospital stay was longer (23 days) and 4 suffered recurrence within one month of discharge. Two patients died after conservative treatment, but it is unclear if this strategy was chosen due to poor general functional status. It was concluded that patients treated with TURP have a shorter length of stay than patients treated with needle aspiration, but the former is a more invasive procedure and carries the risk of greater complications [3]. Another retrospective study examined 48 patients with a prostatic abscess and devised an algorithm for treatment recommendations. For patients with an abscess size <2 cm, minimal symptoms, and no leukocytosis, antibiotics for 2 weeks were suggested. If these criteria were not fulfilled or if there was a persistent abscess, transperineal aspiration was recommended [11].

Of the 21 cases of MRSA prostatic abscess found in the literature, 4 were treated with antibiotics alone and 17 with the combination of antibiotics and at least one additional intervention (TURP, abscess drainage). Two patients died, one who was treated conservatively, and the other with transurethral abscess drainage. The choice of antibiotics, duration, and route of administration have not been well-defined for the treatment of MRSA prostatic abscess. One author has suggested one week of IV vancomycin followed by 4 weeks of trimethoprim and/or rifampin [4]. In our case, vancomycin was given initially and then switched to daptomycin due to ongoing renal injury. We decided to complete 6 weeks of therapy with IV daptomycin for the treatment of MRSA prostatic abscess and bacteremia and had symptomatic, microbiologic, and radiologic resolution. Daptomycin was considered sufficient therapy, as research has shown that it is highly excreted in the urine, has good penetration into biofilms, and is not affected by alkaline pH, which is often seen in prostate tissue [12, 13]. Our case is an example that, even in immunosuppressed patients, a conservative approach can be opted for if adequate follow-up is reassured.

We found 5 case reports of patients with prostatic abscess of any etiology following solid organ transplantation: 4 cases described after kidney transplant and 1 case following heart transplantation. The associated organisms were* Histoplasma capsulatum* [14, 15],* Cladophialophora carrionii* [16],* Pseudomonas aeruginosa* [17], and* Cryptococcus neoformans* [18]. In the cases of the renal transplant patients, the prostatic abscess was found at 2.5 and 3 years (*Histoplasma*), 10 months (*Cladophialophora*), and 6 days (*Pseudomonas*) after transplant. In the heart transplant patient, the abscess was found after 7 years (*Cryptococcus*). To our knowledge, this is the first case describing a prostatic abscess in a liver transplant recipient and the first case of MRSA prostatic abscess described in a solid organ transplant recipient.

## 4. Conclusion

In summary, we present a unique case of a patient with a MRSA prostatic abscess and bacteremia. Abnormal fluctuation of the prostate during rectal examination together with MRSA bacteriuria or MRSA bacteremia of unknown origin should raise the suspicion for this diagnosis. While* S. aureus* is not a common cause of a prostatic abscess, the growing number of these cases in the literature emphasizes the importance of early recognition in susceptible individuals, such as solid organ transplant recipients. Treatment should be individualized based on each patient's clinical condition.

## Figures and Tables

**Table 1:** Reported cases of *Staphylococcus aureus* prostatic abscess.

	Age (years)	Comorbidities/risk factors	Treatment	Microbiology
Our patient	31	DM, OLT	Vancomycin + daptomycin	HA-MRSA (urine, blood)

Deshpande et al., 2013 [19]	49	BPH	Vancomycin + doxycycline, TURP	CA-MRSA (N/A)

Docekal et al., 2013 [5]	56	DM	Vancomycin, IR-guided abscess drainage ×2, pigtail drain placement, TURP	CA-MRSA (prostate abscess)

Krishnamohan et al., 2013 [1]	47	Recent *Trichomonas* infection	Vancomycin + linezolid, TURP, percutaneous and transperineal abscess drainage	CA-MRSA (urine, prostate abscess)
	20	Hepatitis C	Vancomycin + linezolid, TURP	CA-MRSA (prostate abscess)

Lachant et al., 2013 [7]	47	Recent penile furuncle, history of hypospadias with urethral stricture	Vancomycin, TURP	CA-MRSA (urine, blood, lung, heart valve vegetation, kidney, prostate abscess)
	31	None	Vancomycin + daptomycin + TMP-SMX, TURP, transrectal abscess drainage	CA-MRSA (blood, prostate abscess)

Naboush et al., 2013 [4]	52	DM	Vancomycin + TMP-SMX + rifampin, TURP, transurethral unroofing of abscess	CA-MRSA (urine, blood, prostate abscess)

Sukhal et al., 2013 [20]	57	DM	Vancomycin	CA-MRSA (urine, blood, nares)

Flannery and Humphrey 2012 [6]	49	DM	Vancomycin + doxycycline, TURP	CA-MRSA (urine, blood, prostate tissue)

Javeed et al., 2012 [21]	50	DM	Vancomycin + daptomycin, CT-guided abscess drainage	CA-MRSA (urine, blood, prostate abscess)

Matsumoto et al., 2012 [22]	65	DM, indwelling urethral catheter	Antibiotics, transrectal abscess drainage	MSSA (blood, prostate abscess, heart valve vegetation)

Abreu et al., 2011 [23]	59	DM, suppurative lesions in nasal cavity	TMP-SMX + ciprofloxacin + vancomycin, percutaneous abscess drainage	CA-MRSA (blood, prostate abscess)

Park et al., 2011 [24]	45	DM	Vancomycin, percutaneous abscess drainage, TURP	CA-MRSA (blood, prostate abscess)

Tiwari et al., 2011 [25]	N/A	N/A	Intravenous and oral antibiotics, surgical treatment	*S. aureus * ^#^
	N/A	N/A	Intravenous and oral antibiotics, surgical treatment	*S. aureus * ^#^

Chao et al., 2009 [26]	40	N/A	Vancomycin + gentamicin, transperineal abscess drainage	CA-MRSA (blood, prostate abscess)

Baradkar et al., 2008 [27]	50	DM	Ciprofloxacin	MSSA

Gautam et al., 2008 [28]	51	HIV	Ciprofloxacin + TMP-SMX + vancomycin, transurethral abscess drainage	CA-MRSA (blood, prostate abscess)

Lin et al., 2008 [29]	55	BPH	Vancomycin	CA-MRSA (blood)

Pierce et al., 2008 [30]	64	DM	Vancomycin, percutaneous abscess drainage	CA-MRSA (urine, blood, prostate abscess)

Beckman and Edson 2007 [31]	53	DM	Vancomycin + TMP-SMX + rifampin	CA-MRSA (blood, prostate tissue, nares)

Cabrera Meirás et al., 2007 [32]	51	N/A	Antibiotics, transrectal abscess drainage, urinary diversion	*S. aureus * ^#^ (prostate abscess)

Tobian and Obar 2007 [33]	56	DM, BPH, history of self-digital rectal examinations	Vancomycin + rifampin, cystoscopy, percutaneous abscess drainage, transurethral unroofing of abscess	MRSA (urine, blood)∗

Shindel et al., 2006 [34]	29	Straddle injury to urethra, hepatitis C	Vancomycin, Cope loop catheter placement, urethroplasty	CA-MRSA (prostate abscess)

Baker et al., 2004 [35]	43	Hepatitis C, intravenous drug abuse	Vancomycin + nafcillin + TMP-SMX, TURP	CA-MRSA (blood, prostate abscess)

Fraser et al., 2003 [36]	63	DM, recent scrotal abscess drainage	Vancomycin, percutaneous abscess drainage, TURP	HA-MRSA (urine, blood, prostate abscess)

Oliveira et al., 2003 [37]	38	HIV	Intravenous antibiotics, surgical procedure unclear	*S. aureus * ^#^
	41	None	Intravenous antibiotics, surgical procedure unclear	*S. aureus * ^#^
	50	DM	Intravenous antibiotics, surgical procedure unclear	*S. aureus * ^#^
	50	None	Intravenous antibiotics, surgical procedure unclear	*S. aureus * ^#^

Trauzzi et al., 1994 [38]	35	HIV	Intravenous antibiotics	*S. aureus * ^#^

Savarirayan et al., 1995 [39]	50	Recent salivary gland abscess treated with antibiotics	Ciprofloxacin + erythromycin, transperineal abscess drainage, perineal unroofing of abscess	*S. aureus * ^#^

Gill et al., 1991 [40]	42	Recent fall with closed arm fracture	Oxytetracycline, transperineal abscess drainage	MSSA (prostate abscess)

BPH, benign prostate hypertrophy; CA, community-acquired; DM, diabetes mellitus; HA, hospital-acquired; HIV, human immunodeficiency virus; IR, interventional radiology; OLT, orthotopic liver transplant; TURP, transurethral resection of prostate; TMP-SMX, trimethoprim-sulfamethoxazole; MRSA, methicillin-resistant *Staphylococcus aureus*; MSSA, methicillin-sensitive *Staphylococcus aureus*; N/A, not available.

∗Community versus hospital-acquired MRSA status not given.

^#^Susceptibility to methicillin was not reported.
